# Association of Opioids and Sedatives with Increased Risk of In-Hospital Cardiopulmonary Arrest from an Administrative Database

**DOI:** 10.1371/journal.pone.0150214

**Published:** 2016-02-25

**Authors:** Frank J. Overdyk, Oonagh Dowling, Joseph Marino, Jiejing Qiu, Hung-Lun Chien, Mary Erslon, Neil Morrison, Brooke Harrison, Albert Dahan, Tong J. Gan

**Affiliations:** 1 Department of Anesthesiology, Hofstra North Shore-LIJ School of Medicine, New Hyde Park, NY, United States of America; 2 North American Partners in Anesthesia, Melville, NY, United States of America; 3 Department of Medicine, Hofstra North Shore-LIJ School of Medicine, Hempstead, NY, United States of America; 4 Covidien Healthcare Economics and Outcomes Research, Mansfield, MA, United States of America; 5 Covidien Respiratory and Monitoring Solutions, Boulder, CO, United States of America; 6 Harrier Consultancy, Lancaster, United Kingdom; 7 Boulder Medical Writing, Boulder, CO, United States of America; 8 Department of Anesthesiology, Leiden University Medical Center, Leiden, Netherlands; 9 Department of Anesthesiology, Stony Brook University (SUNY), Stony Brook, NY, United States of America; Azienda Ospedaliero-Universitaria Careggi, ITALY

## Abstract

**Background:**

While opioid use confers a known risk for respiratory depression, the incremental risk of in-hospital cardiopulmonary arrest, respiratory arrest, or cardiopulmonary resuscitation (CPRA) has not been studied. Our aim was to investigate the prevalence, outcomes, and risk profile of in-hospital CPRA for patients receiving opioids and medications with central nervous system sedating side effects (*sedatives*).

**Methods:**

A retrospective analysis of adult inpatient discharges from 2008–2012 reported in the Premier Database. Patients were grouped into four mutually exclusive categories: (1) opioids and *sedatives*, (2) opioids only, (3) *sedatives* only, and (4) neither opioids nor *sedatives*.

**Results:**

Among 21,276,691 inpatient discharges, 53% received opioids with or without *sedatives*. A total of 96,554 patients suffered CPRA (0.92 per 1000 hospital bed-days). Patients who received opioids and *sedatives* had an adjusted odds ratio for CPRA of 3.47 (95% CI: 3.40–3.54; p<0.0001) compared with patients not receiving opioids or *sedatives*. Opioids alone and *sedatives* alone were associated with a 1.81-fold and a 1.82-fold (p<0.0001 for both) increase in the odds of CPRA, respectively. In opioid patients, locations of CPRA were intensive care (54%), general care floor (25%), and stepdown units (15%). Only 42% of patients survived CPRA and only 22% were discharged home. Opioid patients with CPRA had mean increased hospital lengths of stay of 7.57 days and mean increased total hospital costs of $27,569.

**Conclusions:**

Opioids and sedatives are independent and additive risk factors for in-hospital CPRA. The impact of opioid sparing analgesia, reduced sedative use, and better monitoring on CPRA incidence deserves further study.

## Introduction

Reducing preventable harm has been a focus of the US healthcare system since the Institute of Medicine report in 1999, and has received renewed attention under the Affordable Care Act, which will not reimburse care associated with certain iatrogenic complications [1]. In spite of a small improvement in outcomes over the past decade, outcomes remain predominantly catastrophic in the approximately 200,000 in-hospital cardiopulmonary arrests (CPA) in the United States, with fewer than 20% of patients surviving to discharge without anoxic brain injury [2–4]. The widespread adoption of Medical Emergency Teams (METs), also known as Rapid Response Teams (RRTs), by hospitals over the last two decades was designed to reduce preventable harm from late recognition of physiologic instability preceding CPA [5, 6].

Opioids remain the preferred analgesics for management of moderate to severe pain among hospitalized patients in the US [7], and the use of opioids has been steadily increasing in the US over the last decade [8–10]. While opioids are highly effective analgesics, unrecognized opioid induced respiratory depression (OIRD) that progresses to cardiopulmonary or respiratory arrest or cardiopulmonary resuscitation (CPRA) is recognized as an important cause of harm [11–14]. In 2012, the Joint Commission, the premier hospital accreditation body in the US, highlighted the risks of opioid therapy through a 2012 Sentinel Event Alert (SEA#49) entitled “Safe Use of Opioids in Hospitals” and proposed strategies for identifying patients at high risk for OIRD while an analysis of a closed claims database for anesthesiologists reinforced the notion that opioid related adverse events are potentially preventable [15, 16]. Along these lines, a recent Premier database analysis by Herzig et al indicated that the majority of hospitalized nonsurgical patients were exposed to opioids and that hospitals that used opioids more frequently had an increased adjusted risk of a severe opioid-related adverse event per patient exposed [17]. Given these concerns, these authors stressed that to improve hospital safety, additional research is needed to further define the predictors of opioid-related adverse events in hospitalized patients [17].

Thus, the primary objective of this study was to investigate an association between opioid therapy and in-hospital CPRA. Since medications with central nervous system depressant effects (*sedatives*) are well known to potentiate the respiratory depressant effects of opioids, we included these medications in the analysis [18]. Recent evidence suggest that benzodiazepines and other medications commonly prescribed alongside opioids to outpatients with chronic pain are significant contributors to preventable harm [19]. A secondary objective was to quantify the proportion of patients who suffered a CPRA who were deemed at low risk for this serious complication, by virtue of their location of care at the time of their CPRA, age, and comorbidities. Lastly, we use this administrative data to project the annual financial burden of CPRA in the US.

## Methods

### Setting and Patients

This was a retrospective analysis of inpatient data from the Premier database (Premier Inc., Charlotte, North Carolina), a privately owned database that represents approximately 1/5^th^ of all US hospitalizations annually [20–22]. The Premier database includes all International Classification of Diseases-9th Revision-Clinical Modification (ICD-9-CM) diagnosis and procedure codes recorded by the hospital, as well as a limited set of Current Procedural Terminology (CPT)-4 codes. Within the database, discharge-level data include information on patient and provider characteristics, diagnoses and procedures, hospital resource utilization, and charges/cost data for all entries, including pharmacy charges. The North Shore-LIJ Health System Institutional Review Board (IRB) determined that IRB approval was not necessary to conduct this study, as data within the Premier database are de-identified in accordance with the Health Insurance Portability and Accountability Act (HIPAA). No patient consent was required for this retrospective analysis.

**Fig 1:**
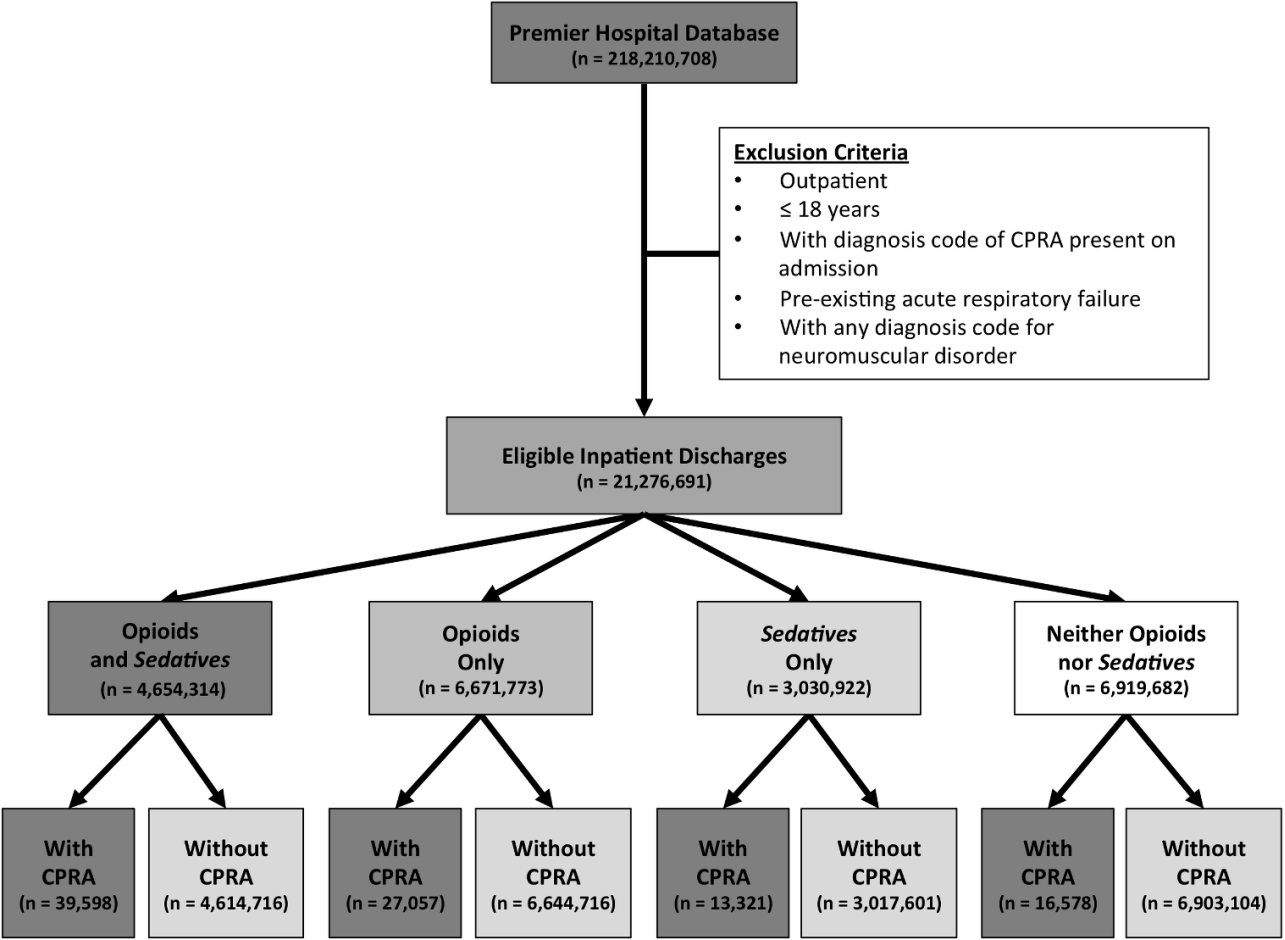
Study Design and Patient Disposition. The Premier Hospital Database was queried for eligible patients who received opioids, with or without medications with sedative properties, and had in-hospital cardiopulmonary or respiratory arrest or cardiopulmonary resuscitation (CPRA) as defined through ICD-9-CM and CPT codes.

Patients were included in the analysis if: (1) they were an adult (>18 years) inpatient from a Premier database hospital and (2) they were hospitalized for at least 1 night between 01 January 2008 and 31 December 2012 (Fig 1). Outcomes of interest were patients who had a diagnosis or procedural code for CPA (ICD-9 = 427.5), respiratory arrest (RA, ICD-9 = 799.1), or cardiopulmonary resuscitation (CPR, ICD-9 procedure = 99.60 or CPT = 92950) entered into their billing record. Patients meeting these diagnostic criteria were included in our definition of CPRA. Patients were excluded if they: (1) were outpatients, (2) had an admitting diagnosis of CPRA (based on ICD-9-CM codes), (3) had pre-existing acute respiratory failure based on the presence on admission of ICD-9 518.81 and 518.84 as the principal diagnosis or secondary diagnosis, or (4) had a diagnosis of a neuromuscular disorder (S1 Table).

Four mutually exclusive groups were identified based on patient medication records, with patients receiving: (1) opioids and *sedatives*, (2) opioids only, (3) *sedatives* only, and (4) neither opioids nor *sedatives* (S2 Table). The hospital location of care (LOC) at the time of CPRA was determined from the location of CPR or the location in which resuscitation drugs (epinephrine, vasopressin, or amiodarone) were administered. For patients who had RA only, administration of naloxone (with or without the above drugs) was used to define event location. Unadjusted clinical and cost outcomes were compared in admissions with CPRA vs. those without CPRA in the cohort of patients who had received opioids during admission. As quantifying CPRA preventable harm in low risk patients was one of our goals, we created a ‘low risk’ category, which we arbitrarily but logically assigned to patients younger than 61 years of age *with* a low Charlson Comorbidity Index (CCI = 0 or 1) *and/or* an All Patients Refined Severity of Illness (APR) score categorized as ‘minor’ or ‘moderate’ (APR = 1 or 2, respectively).

### Statistical Analysis

Descriptive statistics (principally prevalence percentages for categorical variables and mean and standard deviation [SD] for continuous variables) were used to compare patient characteristics. Two-sample t-tests and one-way analysis of variance (ANOVA) were used to compare continuous variables and chi-square tests were used to compare categorical variables. Multivariate logistic regression was used to estimate the odds ratio for the occurrence of CPRA, adjusting for patient age, gender, race, CCI, comorbid conditions, medical vs. surgical patient, admission type, and hospital type. Results were expressed as odds ratio (OR) and 95% confidence intervals (CI). All statistical analyses were conducted using SAS® 9.2 for UNIX (Cary, NC, USA).

## Results

### Incidence

Overall, 21,276,691 inpatient discharges between 2008 and 2012 were eligible for this analysis (Fig 1). Of these, 4,654,314 (21.9%) received opioids and *sedatives*, 6,671,773 (31.4%) received opioids only, 3,030,922 (14.2%) received *sedatives* only, and 6,919,682 (32.5%) received neither opioids nor *sedatives*. CPRA was identified in 96,554 discharges, for an overall CPRA rate of 4.54 cases per 1000 hospital admissions, or an average of 0.92 (Interquartile range: 0.56–1.15) cases per 1000 inpatient hospital-days. Patients who received opioids and *sedatives* showed the highest rate of in-hospital CPRA (n = 39,598 [0.85%]) when compared to opioids only (n = 27,057 [0.41%]), *sedatives* only (n = 13,321 [0.44%]), and neither opioids nor *sedatives* (n = 16,578 [0.24%]). Among the 96,544 CPRA cases, cardiac arrest was most commonly coded (n = 54,618 [56.6%]), followed by CPR only (n = 36,319 [37.6%]), and RA only (n = 5,005 [5.2%]). A small number of patients were coded as both CPA and RA (n = 612 [0.6%]) (S3 Table).

### Patient Demographics

All four groups (opioids and *sedatives*, opioids only, *sedatives* only, and neither opioids nor *sedatives*) were similar with respect to demographic and clinical characteristics, except that patients receiving opioids were more likely to be electively admitted and receive surgical intervention, compared with those not receiving opioids (Table 1). Patients with CPRA tended to be older (66.7 vs. 57.2 years), have a higher comorbidity index (3.12 vs. 1.55), and were less likely to be elective admissions (16.1% vs. 28.1%) than patients without CPRA (Table 2).

**Table 1:** Overall Patient Demographics.

Characteristic	Opioids and *Sedatives* (n = 4,654,314)	Opioids Only (n = 6,671,773)	*Sedatives* Only (n = 3,030,922)	Neither Opioids nor *Sedatives* (n = 6,919,682)
**Age**				
Overall	56.45 ± 18.02	54.80 ± 20.33	59.45 ± 19.87	59.27 ± 22.03
> 80 years	478,176 (10.3)	821,750 (12.3)	537,386 (17.7)	1,518,909 (22.0)
71–80 years	666,531 (14.3)	961,930 (14.4)	522,571 (17.2)	1,200,953 (17.4)
61–70 years	864,886 (18.6)	1,088,622 (16.3)	496,521 (16.4)	992,291 (14.3)
51–60 years	928,515 (20.0)	1,025,155 (15.4)	476,302(15.7)	803,553 (11.6)
18–50 years	1,716,206 (36.9)	2,774,316 (41.6)	998,142 (32.9)	2,403,976 (34.7)
**Gender**				
Male	1,802,136 (38.7)	2,538,768 (38.1)	1,340,637 (44.2)	2,786,071 (40.3)
Female	2,851,805 (61.3)	4,132,602 (61.9)	1,690,053 (55.8)	4,133,201 (69.7)
Unknown	373 (<0.1)	403 (<0.1)	232 (<0.1)	410 (<0.1)
**Race**				
White	3,263,492 (70.1)	4,239,220 (63.5)	2,084,510 (68.8)	4,259,130 (61.6)
Black	561,773 (12.1)	934,830 (14.0)	376,288 (12.4)	980,147 (14.2)
Hispanic	155,867 (3.4)	293,853 (4.4)	110,629 (3.7)	322,979 (4.7)
Other	673,182 (14.5)	1,203,870 (18.0)	459,495 (15.2)	1,357,426 (19.6)
**CCI**				
Overall	1.76 ± 2.31	1.40 ± 2.08	1.62 ± 2.00	1.54 ± 1.94
> 2	1,159,306 (24.9)	1,315,573 (19.7)	731,330 (24.1)	1,620,750 (23.4)
2	666,383 (14.3)	829,426 (12.4)	449,831 (14.8)	986,786 (14.3)
1	981,263 (21.1)	1,257,363 (18.9)	686,578 (22.7)	1,481,655 (21.4)
0	1,847,362 (39.7)	3,269,411 (49.0)	1,163,183 (38.4)	2,830,491 (40.9)
**APR Severity of Illness**				
Extreme	488,171 (10.5)	345,794 (5.2)	188,246 (6.2)	281,476 (4.1)
Major	1,279,411 (27.5)	1,448,978 (21.7)	866,921 (28.6)	1,825,195 (26.4)
Moderate	1,733,521 (37.3)	2,486,885 (37.3)	1,323,638 (43.7)	2,776,858 (40.1)
Minor	1,153,211 (24.8)	2,390,116 (35.8)	652,117 (21.5)	2,036,153 (29.4)
**Patient Type**				
Medical	2,448,268 (52.6)	3,184,007 (47.7)	2,619,567 (86.4)	6,252,967 (90.4)
Surgical	2,206,046 (47.4)	3,487,766 (52.3)	411,355 (13.6)	666,715 (9.6)
**Admission Type**				
Non-elective	3,203,731 (68.8)	4,173,683 (62.6)	2,468,080 (81.4)	5,466,537 (79.0)
Elective	1,450,583 (31.2)	2,498,090 (37.4)	562,842 (18.6)	1,453,145 (21.0)
**Teaching Hospital**				
Yes	1,786,899 (38.4)	2,731,100 (40.9)	1,148,914 (37.9)	2,786,882 (40.3)
No	2,867,415 (61.6)	3,940,673 (59.1)	1,882,008 (62.1)	4,132,800 (59.7)
**Hospital Bed Size**				
> 500	1,450,216 (31.2)	2,098,363 (31.5)	838,960 (27.7)	1,870,465 (27.0)
250–500	2,113,139 (45.4)	3,088,380 (46.3)	1,430,406 (47.2)	3,356,746 (48.5)
< 250	1,090,959 (23.4)	1,485,030 (22.3)	761,556 (25.1)	1,692,471 (24.5)
**Region**				
South	2,260,017 (48.6)	2,682,391 (40.2)	1,350,586 (44.6)	2,502,339 (36.2)
Northeast	668,478 (14.4)	1,406,663 (21.1)	532,299 (17.6)	1,783,244 (25.8)
Midwest	832,971 (17.9)	1,258,154 (18.9)	648,674 (21.4)	1,514,790 (21.9)
West	892,848 (19.2)	1,324,565 (19.9)	499,363 (16.5)	1,119,309 (16.2)
**Hospital Location**				
Rural	558,631 (12.0)	640,906 (9.6)	394,179 (13.0)	792,015 (11.5)
Urban	4,095,683 (88.0)	6,030,867 (90.4)	2,636,743 (87.0)	6,127,667 (88.6)

Values presented as mean ± SD and n (column %). P-values < 0.0001 for all comparisons. APR = All Patient Refined Severity of Illness score (http://solutions.3m.com/wps/portal/3M/en_US/Health-Information-Systems/HIS/Products-and-Services/Products-List-A-Z/APR-DRG-Software/); CCI = Charlson Comorbidity Index.

**Table 2:** Patient Demographics with and without CPRA.

Characteristic	With CPRA (n = 96,554)	Without CPRA (n = 21,180,137)
**Age**		
Overall	66.7 ± 16.6	57.2 ± 20.5
18–50 years	15,737 (16.3)	7,876,903 (37.2)
51–60 years	14,814 (15.3)	3,218,711 (15.2)
61–70 years	19,999 (20.7)	3,422,321 (16.2)
71–80 years	23,174 (24.0)	3,328,811 (15.7)
>80 years	22,830 (23.6)	3,333,391 (15.7)
**Gender**		
Male	53,183 (55.1)	8,414,429 (39.7)
Female	43,366 (44.9)	12,764,295 (60.3)
Unknown	5 (<0.1)	1,413 (<0.1)
**CCI**		
Overall	3.12 ± 2.56	1.55 ± 2.08
0	13,939 (14.4)	9,096,508 (43.0)
1	16,054 (16.6)	4,390,805 (20.7)
2	15,652 (16.2)	2,916,774 (13.8)
>2	50,909 (52.7)	4,776,050 (22.6)
**APR Severity of illness**		
Minor	3,274 (3.4)	6,228,323 (29.4)
Moderate	8,581 (8.9)	8,312,321 (39.3)
Major	23,707 (24.6)	5,396,798 (25.5)
Extreme	60,992 (63.2)	1,242,695 (5.9)
**Race**		
White	58,578 (60.7)	13,787,774 (65.1)
Black	18,128 (18.8)	2,834,910 (13.4)
Hispanic	3,408 (3.5)	879,920 (4.2)
Other	16,440 (17.0)	3,677,533 (17.4)
**Patient type**		
Medical	54,745 (56.7)	14,450,064 (68.2)
Surgical	41,809 (43.3)	6,730,073 (31.8)
**Admission type**		
Elective	15,514 (16.1)	5,949,146 (28.1)
Non-elective	81,040 (83.9)	15,230,991 (71.9)
**Teaching Hospital**		
Yes	41,460 (42.9)	8,412,335 (39.7)
No	55,094 (57.1)	12,767,802 (60.3)
**Hospital Bed Size**		
>500	29,974 (31.0)	6,228,030 (29.4)
250–500	46,805 (48.5)	9,941,866 (46.9)
<250	19,775 (20.5)	5,010,241 (23.7)
**Region**		
South	42,858 (44.4)	8,752,475 (41.3)
Northeast	17,197 (17.8)	4,373,487 (20.7)
Midwest	18,241 (18.9)	4,236,348 (20.0)
West	18,258 (18.9)	3,817,827 (18.0)
**Hospital Location**		
Urban	86,343 (89.4)	18,804,617 (88.8)
Rural	10,211 (10.6)	2,375,520 (11.2)

Values presented as mean ± SD and n (column %). P-values < 0.0001 for all comparisons. APR = All Patient Refined Severity of Illness score (http://solutions.3m.com/wps/portal/3M/en_US/Health-Information-Systems/HIS/Products-and-Services/Products-List-A-Z/APR-DRG-Software/); CCI = Charlson Comorbidity Index; CPRA = cardiopulmonary or respiratory arrest or cardiopulmonary resuscitation.

### Opioid/*Sedative* Use and the Risk of In-Hospital CPRA

**Fig 2:**
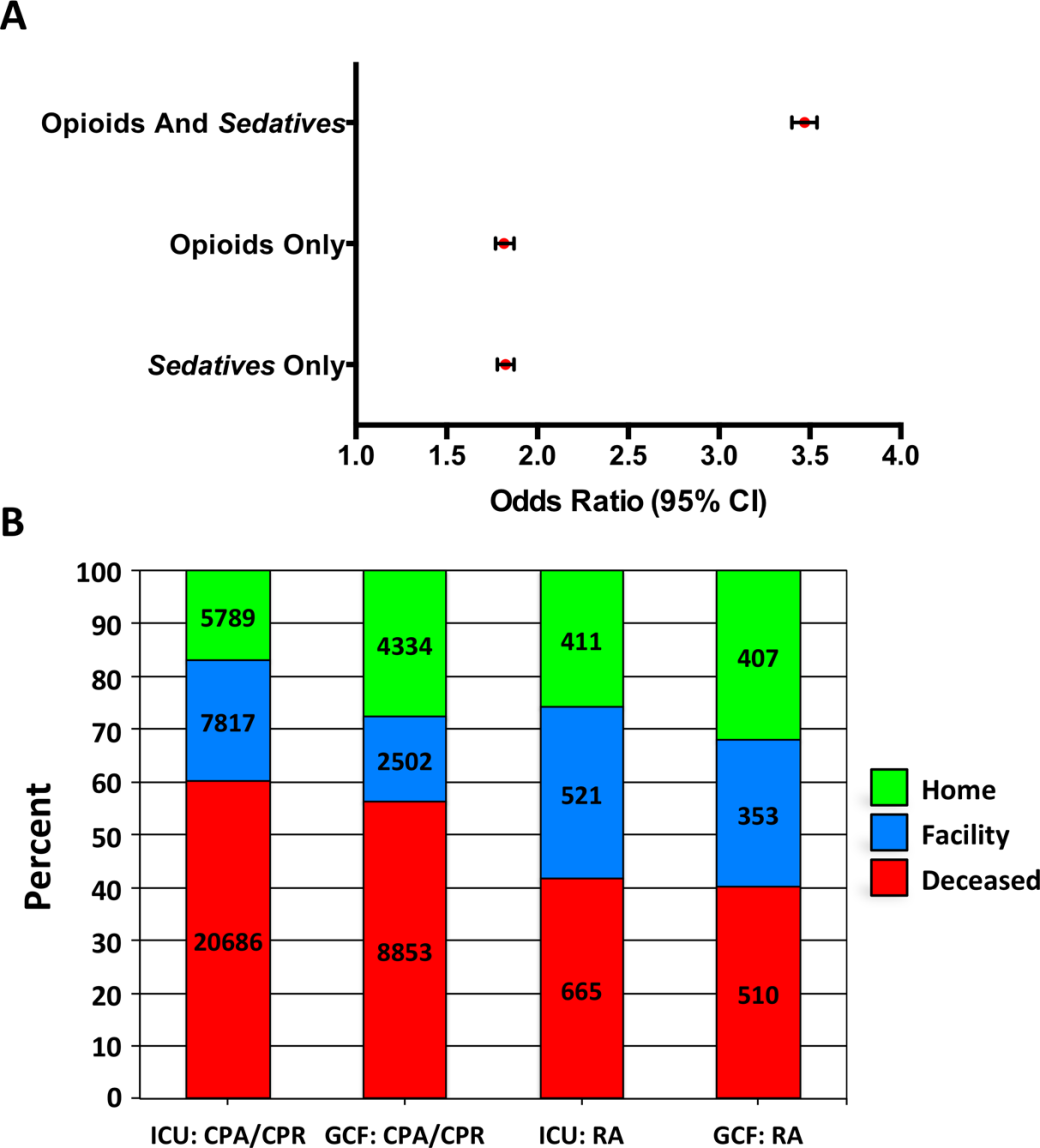
Odds Ratios and Opioid Patient Disposition. **(A)** Adjusted Odds Ratio (95% CI) of cardiopulmonary or respiratory arrest or cardiopulmonary resuscitation (CPRA) by medication type. **(B)** Disposition (%) of opioid patients by location of arrest. The number of patients per category is shown within each bar. Facility: includes skilled nursing facility; intermediate care facility; hospice-medical facility; swing bed; another rehab facility; long-term care hospital; nursing facility; hospice-home; federal hospital or critical access hospital. CPA = cardiopulmonary arrest; CPR = cardiopulmonary resuscitation; GCF = general care floor; ICU = intensive care unit; RA = respiratory arrest.

Opioids and *sedatives* were independent and additive risk factors for in-hospital CPRA (Fig 2A and S4 Table). For patients who received opioids and *sedatives*, the odds of in-hospital CPRA were increased 3.47-fold (95% CI: 3.40–3.54; p<0.0001) (Fig 2A and S4 Table). For patients who received opioids alone, the odds of CPRA were increased 1.81-fold (95% CI: 1.77–1.85; p<0.0001). Similarly, the odds of in-hospital CPRA for patients who received *sedatives* only were increased 1.82-fold (95% CI: 1.78–1.87; p<0.0001).

### Outcomes, Discharge Location, and Acuity

The unadjusted survival to discharge rates for opioid recipients (with and without *sedatives*) were 42.1% for patients with CPRA and 98.0% for patients without CPRA (Table 3). For patients receiving opioids, the mortality rates were significantly higher for patients who experienced CPA and/or CPR (CPA/CPR; 58.8%) as compared to patients who experienced RA only (39.3%; p<0.0001) (S5 Table).

**Table 3:** Outcome Analysis in Opioid Patients: Discharge Disposition, Length of Hospital Stay, and Cost of Care.

Variable	With CPRA (n = 66,655)	Without CPRA (n = 11,259,432)	Unadjusted Mean Difference
**Survival to Discharge**	28,077 (42.1)	11,029,436 (98.0)	(-55.9)
**Discharge Status**			
Deceased	38,500 (57.8)	219,977 (2.0)	(55.8)
Facility	13,715 (20.6)	1,965,179 (17.5)	(3.1)
Home	14,362 (21.6)	9,064,257 (80.5)	(-58.9)
**Hospital Stay**			
Length of Stay	12.54 (18.52)	4.97 (8.18)	7.57
ICU stay	8.03 (11.10)	3.83 (6.21)	4.20
**Hospital Cost**			
Total hospital cost	$40,354 ($56,338)	$12,785 ($18,866)	$27,569
Room and board cost	$16,531 ($26,636)	$4,707 ($8,211)	$11,824
Surgery cost	$3,641 ($7,048)	$2,552 ($4,518)	$1,089
Central supply cost	$4,758 ($9,702)	$2,582 ($8,009)	$2,176
Anesthesia cost	$716 ($1,600)	$459 ($683)	$257
Pharmacy cost	$5,094 ($12,499)	$1,183 ($6,406)	$3,911
Other cost	$11,161 ($17,238)	$2,963 ($4,743)	$8,198
**ICU cost**	$13,943 ($21,651)	$6,349 ($10,777)	$7,594

Values presented as mean (SD) days or $US, and n (column %). CPRA = cardiopulmonary or respiratory arrest or cardiopulmonary resuscitation; ICU = intensive care unit.

In the 62,811 patients who received opioids with or without *sedatives* whose LOC (intensive care unit [ICU], stepdown unit, or general care floor [GCF]) at the time of the CPRA was known, 59,316 (94.4%) suffered CPA/CPR whereas only 3,495 (5.6%) suffered RA (S5 Table). When mortality rates following CPRA for opioid patients were analyzed by LOC, 20,686 (60.3%) patients with CPA/CPR in the ICU died, 8,853 (56.4%) patients with CPA/CPR on the GCF died, 665 (41.6%) patients with RA in the ICU died, and 510 (40.1%) patients with RA on the GCF died (Fig 2B and S5 Table). While 21,351 (55.4%) of the opioid patients who died following CPRA were in the ICU, there were also 9,363 (24.3%) opioid patients who died after CPRA on the GCF (S5 Table). Opioid patients with RA only had higher discharge rates to home or an extended care facility, and lower death rates, than patients with CPA/CPR (Fig 2B and S5 Table). For opioid patients with a known LOC at the time of CPRA, the care setting for RA patients was somewhat evenly divided into the ICU (45.7%) and the GCF (36.4%) whereas the LOC for CPA/CPR patients was predominantly the ICU (57.9% vs. 26.5% GCF) (S5 Table). In the CPRA group, we were unable to categorize the CPRA location in 8,325 (8.6%) patients and disposition in 122 (0.1%) patients.

By our definition, 7,508 (0.07%) low risk patients (age < 61 years *with* CCI = 0,1 *and/or* APR = 1,2) receiving opioids suffered CPA/CPR in the ICU (n = 3,825) or on the GCF (n = 3,683) during the study period, whereas 1,507 (0.02%) low risk without opioid patients suffered CPA/CPR in the ICU (n = 798) or on the GCF (n = 709). Additionally, 446 (0.004%) low risk opioid patients suffered RA in the ICU (n = 227) or on the GCF (n = 219), while 45 (0.0005%) low risk without opioid patients suffered RA in the ICU (n = 30) or on the GCF (n = 15) (S6 Table).

### Length of Stay and Cost of Care

Among opioid patients, CPRA increased the mean length of hospital stay by 7.57 days, and increased the mean length of ICU stay by 4.20 days (Table 3). Admissions in which an opioid patient developed CPRA were associated with significantly higher costs than admissions without CPRA, with an unadjusted mean increase in total hospital costs of $27,569 and an increase in ICU costs of $7,594 (Table 3). Based on our study cohort, we projected a national incidence of 280,883 CPRA cases between 2008 and 2012 (S7 Table). With a projected average cost difference of $27,119 per CPRA patient, CPRA may contribute up to $1.5 billion per year in inpatient hospitalization costs.

## Discussion

Opioid analgesic therapy was associated with an almost 2-fold increased risk of CPA, adjusted for confounding variables, and this risk was increased to 3.5-fold when patients received central nervous system sedating medications in addition to opioids. Despite our knowledge that sedatives are known to potentiate the effect of opioids, the magnitude of the incremental risk of cardiopulmonary arrest for patients receiving sedatives was unexpected. In this nationally representative sample of almost 21 million hospitalized adults in the US, CPRA was associated with an increased risk of death, prolonged hospital stay, and increased cost of care in patients receiving opioids and sedatives versus the same outcome (CPRA) in patients receiving neither.

Our CPRA rate of 0.92 per 1000 hospital bed-days is the same as that reported by Merchant et al in 2011 using the Get With The Guidelines-Resuscitation (GWTG-R) registry, though, in contrast to the Merchant study, our analysis included a relatively small number of RA patients [23]. Among opioid patients, we identified mortality rates of 57.9% for CPRA and 58.8% for CPA/CPR. These mortality rates are lower than those reported in the National Registry of Cardiopulmonary Resuscitation from 2000 to 2002 (83%) and in the GWTG-R registry between 2000 and 2009 (for asystole or pulseless electrical activity; also 83%), though differences in study populations and analysis parameters, such as our inclusion of RA in isolation, make direct comparisons across studies difficult [3, 24]. As expected, the mortality rate for RA only in our patient cohort (39.3%) was significantly lower than that of CPA/CPR. Consistent with previous reports, our analysis indicated that CPRA patients were more likely to be older, male, and African-American patients, with a higher comorbidity index [25–28].

Our findings are consistent with the persistently high prevalence of the patient safety indicator (PSI)—death among surgical inpatients with serious treatable complications, formerly known as ‘failure to rescue’ [29]. PSIs are defined by the AHRQ (Agency for HealthCare Quality and Research) as potentially preventable complications and other iatrogenic events resulting from exposure to the health care system, and CPRA is one of the most prominent. Hillman et al identified vital sign abnormalities at least 8 hours prior to ICU admission or in-hospital cardiac arrest that were deemed preventable with earlier intervention [30]. In an effort to reduce ‘failure to rescue events’, the RRT/MET concept was implemented in the early 2000s with the intent to reduce delayed recognition of clinical decompensation in hospitalized patients. RRTs were quickly deployed in Health Systems worldwide despite consistent evidence that they could reliably reduce in-hospital morbidity and mortality [6, 31]. However, Galhotra et al. concluded from an analysis of a ‘mature’ RRT that increased monitoring and improved adherence to hospital patient care policies are essential to reducing potentially avoidable CPRA [32]. A subsequent consensus conference on the afferent limb of the RRT system reaffirmed that timely detection of patients who experience physiologic decompensation, as opposed to the timely response of the RRT team (efferent limb), remains a major weakness in the RRT system [33]. This conclusion was recently supported by continuous oximetry monitoring data from the Cleveland Clinic that showed that over a third of postsurgical patients experienced prolonged periods of hypoxia (SpO_2_ <90%), unbeknownst to their bedside providers [34].

The association of opioids with CPRA is most likely multifactorial, and is logically stronger in surgical patients and those with complex comorbidities. However, in ‘low risk’ patients on the GCF, undetected critical respiratory depression from opioids and sedatives may account for a greater proportion of CPRA events than in ICU patients. As noted above, the magnitude of the incremental risk of cardiopulmonary arrest for patients receiving sedatives was unexpected. The diverse mechanism of action of drugs with sedative properties included in our analysis, however, precludes us from drawing any specific conclusions regarding the incremental risk from any particular family of sedatives.

Current monitoring intervals of vital signs on the GCF as far as 4 hours apart may fail to detect respiratory compromise, and intermittent, manual respiratory rates have been shown to be inaccurate and unreliable [35]. Although continuous, electronic monitoring with pulse oximetry via central telemetry reduced transfers to the ICU and RRT interventions, the potential for alarm fatigue and the financial burden of implementing continuous electronic monitoring remain significant barriers to its widespread adoption [36]. Recently, the Center for Clinical Standards and Quality recommended more provider education on the proper dosing, administration, and identification of patients at high risk for OIRD, and improved monitoring [37]. Our data support the earlier work that outcomes may be improved from earlier detection of respiratory compromise, before patient decompensation escalates to the level of CPRA.

The use of data from an administrative database has both strengths and weaknesses. Clearly, chart review of clinical events recorded through a rigorous and validated methodology, such as those in the GWTG-R registry, is highly reliable. The weaknesses of administrative databases, including coding errors of commission and omission, are well documented. However, it is unlikely that errors of commission in coding events with serious implications such as death and CPRA will go undetected. More likely are errors of omission in drug administered, although as controlled substances, opioids and sedatives are tightly tracked with audit trails, and the billing/coding database is linked to the pharmacy database. Administrative databases neither capture the clinical care rendered, such as vital signs and provider notes that might have confirmed our primary outcomes, nor do they capture a detailed chronology of events. For example, patients in our cohort may have received opioids and sedatives *only after* their CPRA for sedation and analgesia [38], but we are encouraged by the similarity of our outcomes with previous literature on CPA and RA, derived from the GTWG registry and other validated sources. We also note that the associations identified by our analysis may be diluted in countries where the use of opioids and medications with sedative properties is less prevalent.

In conclusion, opioids and sedatives are independent risk factors for in-hospital CPRA, and the additional risk from concomitant administration of medications with sedative properties is significant. Although we report an association and cannot demonstrate causation, we believe this analysis supports concerns that undetected respiratory depression from the synergistic effect of opioids and sedatives may cause significant harm, a portion of it preventable. Further study into the impact of opioid sparing multimodal analgesia, judicious use of sedating co-medications, and appropriate use of continuous electronic monitoring in low acuity settings on CPRA is warranted.

## Supporting Information

S1 Table Exclusionary ICD-9-CM codes. (DOCX)

S2 Table Types of Medications Included in the Study. (DOCX)

S3 Table Coding Filter Distributions. (DOCX)

S4 Table Opioid/*Sedative* Use and Adjusted Risk of In-Hospital CPRA. (DOCX)

S5 Table Association between Opioid/*Sedative* Use, Location of Arrest, and Patient Outcome (Discharge Status). (DOCX)

S6 Table Opioid/*Sedative* Use, Location of Arrest, and Patient Acuity. (DOCX)

S7 Table Association between CPRA and Length of Hospital Stay and Cost of Care In Patients Receiving Opioids Projected Nationally in US. (DOCX)
