# Supplementary material for: Association of Opioids and Sedatives with Increased Risk of In-Hospital Cardiopulmonary Arrest from an Administrative Database
Source: PLoS One. 2016 Feb 25;11(2):e0150214. doi: 10.1371/journal.pone.0150214 (PMC4767404; doi:10.1371/journal.pone.0150214)
Supplement: S1 Table — (DOCX) [file pone.0150214.s001.docx]

**S1 Table. Exclusionary ICD-9-CM codes.**

| **ICD-9-CM** | **Neuromuscular Disorders** |
| --- | --- |
| 357.0 | AC INFECT POLYNEURITIS |
| 358.00 | MYSTHNA GRVS W/O AC EXAC |
| 358.01 | MYASTHNA GRAVS W AC EXAC |
| 358.1 | MYASTHENIA IN OTH DIS |
| 358.2 | TOXIC MYONEURAL DISORDER |
| 358.8 | MYONEURAL DISORDERS NEC |
| 358.9 | MYONEURAL DISORDERS NOS |
| 359.0 | CONG HERED MUSC DYSTRPHY |
| 359.1 | HERED PROG MUSC DYSTRPHY |
| 359.2 | MYOTONIC DISORDERS |
| 359.22 | MYOTONIA CONGENITA |
| 359.23 | MYOTONIC CHONDRODYSTRPHY |

NEC = not elsewhere classified; NOS = not otherwise specified; OTH = other
