# Supplementary material for: Association of Opioids and Sedatives with Increased Risk of In-Hospital Cardiopulmonary Arrest from an Administrative Database
Source: PLoS One. 2016 Feb 25;11(2):e0150214. doi: 10.1371/journal.pone.0150214 (PMC4767404; doi:10.1371/journal.pone.0150214)
Supplement: S2 Table — (DOCX) [file pone.0150214.s002.docx]

**S2 Table. Types of Medications Included in the Study.**

| **Opioids** | ***Sedatives*** | |
| --- | --- | --- |
| Alfentanil hydrochloride  Codeine  Fentanyl  Fentanyl citrate  Hydrocodone bitartrate  Hydromorphone  Levorphanol  Loperamide  Meperidine  Methadone  Morphine  Oxycodone  Propoxyphene  Remifentanil  Sufentanil  Tramadol | Anticonvulsants  Gabapentin  Pregabalin  Antiemetics  Diphenhydramine  Promethazine  Transdermal scopolamine  Dimenhydrinate  Prochlorperazine  Benzodiazepines  Triazolam  Temazepam  Lorazepam  Diazepam  Clonazepam | Muscle Relaxants  Cyclobenzaprine  Opioid Antagonists  Naltrexone  Nalmefene  Sleep aids  Zolpidem  Eszopiclone  Ramelteon  Tricyclic antidepressants  Amitriptyline  Imipramine  Doxepin  Nortriptyline |
