# Supplementary material for: Association of Opioids and Sedatives with Increased Risk of In-Hospital Cardiopulmonary Arrest from an Administrative Database
Source: PLoS One. 2016 Feb 25;11(2):e0150214. doi: 10.1371/journal.pone.0150214 (PMC4767404; doi:10.1371/journal.pone.0150214)
Supplement: S3 Table — (DOCX) [file pone.0150214.s003.docx]

**S3 Table. Coding Filter Distributions.**

| **Filter** | **Opioids and *Sedatives*** (n=4,654,314) | **Opioids Only** (n=6,671,773) | ***Sedatives* Only** (n=3,030,922) | **Neither Opioids nor *Sedatives*** (n=6,919,682) |
| --- | --- | --- | --- | --- |
| No CPRA Filter | 4,614,716 (99.15) | 6,644,716 (99.59) | 3,017,601 (99.56) | 6,903,104 (99.76) |
| CPRA Filter | 39,598 (0.85) | 27,057 (0.41) | 13,321 (0.44) | 16,578 (0.24) |
| Cardiac Arrest Only  (ICD-9-CM 427.5) | 23,520 (0.51) | 14,786 (0.22) | 7,441 (0.25) | 8,871 (0.13) |
| CPR Only  (ICD-9-CM 99.60 and/or CPT 92950) | 13,368 (0.29) | 10,893 (0.16) | 5,066 (0.17) | 6,992 (0.10) |
| Respiratory Arrest Only  (ICD-9-CM 799.1) | 2,430 (0.05) | 1,230 (0.02) | 725 (0.02) | 620 (0.01) |
| Both Cardiac Arrest and Respiratory Arrest | 280 (0.01) | 148 (0.00) | 89 (0.00) | 95 (0.00) |

Values presented as n (column %). CPRA = cardiopulmonary or respiratory arrest; CPR = cardiopulmonary resuscitation.
