# Supplementary material for: Association of Opioids and Sedatives with Increased Risk of In-Hospital Cardiopulmonary Arrest from an Administrative Database
Source: PLoS One. 2016 Feb 25;11(2):e0150214. doi: 10.1371/journal.pone.0150214 (PMC4767404; doi:10.1371/journal.pone.0150214)
Supplement: S4 Table — (DOCX) [file pone.0150214.s004.docx]

**S4 Table. Opioid/*Sedative* Use and Adjusted Risk of In-Hospital CPRA.**

| **Medication Use** | **With CPRA** | **Without CPRA** | **Adjusted Odds Ratio** | **P-value** |
| --- | --- | --- | --- | --- |
| Opioids and *Sedatives* | 39,598 (41.0) | 4,614,716 (21.8) | 3.47 (3.40, 3.54) | <0.0001 |
| Opioids only | 27,057 (28.0) | 6,644,716 (31.4) | 1.81 (1.77, 1.85) | <0.0001 |
| *Sedatives* only | 13,321 (13.8) | 3,017,601 (14.3) | 1.82 (1.78, 1.87) | <0.0001 |
| Neither Opioids nor *Sedatives* | 16,578 (17.2) | 6,903,104 (32.6) | Ref. |  |

Values presented as n (column %) or adjusted odds ratio (95% CI). CPRA = cardiopulmonary or respiratory arrest.
