# Supplementary material for: Association of Opioids and Sedatives with Increased Risk of In-Hospital Cardiopulmonary Arrest from an Administrative Database
Source: PLoS One. 2016 Feb 25;11(2):e0150214. doi: 10.1371/journal.pone.0150214 (PMC4767404; doi:10.1371/journal.pone.0150214)
Supplement: S5 Table — (DOCX) [file pone.0150214.s005.docx]

**S5 Table. Association between Opioid/*Sedative* Use, Location of Arrest, and Patient Outcome (Discharge Status).**

| **Arrest Type** |  | **Opioids +/- *Sedatives*** | | | |  | **Neither Opioids nor *Sedatives*** | | | |
| --- | --- | --- | --- | --- | --- | --- | --- | --- | --- | --- |
|  | **Location** | **Deceased** | **Facility** | **Home** | **Other** | **Location** | **Deceased** | **Facility** | **Home** | **Other** |
| **CPA/**  **CPR** | **Overall** (n=62,995) | 37,061 (58.8) | 13,289 (21.1) | 12,571 (20.0) | 74 (0.1) | **Overall** (n=15,958) | 11,704 (73.3) | 2,701 (16.9) | 1,531 (9.6) | 22 (0.1) |
|  | **ICU** (n=34,332) | 20,686 (60.3) | 7,817 (22.8) | 5,789 (16.9) | 40 (0.1) | **ICU**  (n=5,472) | 4,153 (75.9) | 581 (10.6) | 731 (13.4) | 7 (0.1) |
|  | **Stepdown** (n=9,272) | 4,805 (51.8) | 1,762 (19.0) | 2,695 (29.1) | 10 (0.1) | **Stepdown** (n=2,920) | 1,807 (61.9) | 302 (10.3) | 809 (27.7) | 2 (0.1) |
|  | **GCF** (n=15,712) | 8,853 (56.4) | 2,502 (15.9) | 4,334 (27.6) | 23 (0.2) | **GCF** (n=4,546) | 3,100 (68.2) | 424 (9.3) | 1015 (22.3) | 7 (0.2) |
|  | **Other** (n=3,679) | 2,717 (73.9) | 490 (13.3) | 471 (12.8) | 1 (0.0) | **Other** (n=3,020) | 2,644 (87.6) | 224 (7.4) | 146 (4.8) | 6 (0.2) |
| **RA** | **Overall** (n=3,660) | 1,439 (39.3) | 1,073 (29.3) | 1,144 (31.3) | 4 (0.1) | **Overall** (n=620) | 334 (53.9) | 134 (21.6) | 151 (24.4) | 1 (0.2) |
|  | **ICU**  (n=1,598) | 665 (41.6) | 521 (32.6) | 411 (25.7) | 1 (0.1) | **ICU**  (n=191) | 114 (59.7) | 39 (20.4) | 38 (19.9) | 0 (0.0) |
|  | **Stepdown** (n=625) | 200 (32.0) | 209 (33.4) | 215 (34.4) | 1 (0.2) | **Stepdown** (n=144) | 68 (47.2) | 41 (28.5) | 35 (24.3) | 0 (0.0) |
|  | **GCF** (n=1,272) | 510 (40.1) | 353 (27.8) | 407 (32.0) | 2 (0.2) | **GCF**  (n=219) | 116 (53.0) | 50 (22.8) | 52 (23.7) | 1 (0.5) |
|  | **Other** (n=165) | 64 (38.8) | 61 (37.0) | 40 (24.2) | 0 (0.0) | **Other**  (n=66) | 36 (54.6) | 21 (31.8) | 9 (13.6) | 0 (0.0) |

Values presented as n (row %). For this analysis, patient discharge status such as ”Still a patient “or “information not available” are grouped as “Other”. For Location of Care (LOC) analysis, room and Board charges from “Psychiatric”, “Rehabilitation”, “Miscellaneous”, “Skilled Nursing Facility”, “Detoxification”, or missing care unit information are grouped as “Other”. CPA = cardiopulmonary arrest; CPR = cardiopulmonary resuscitation; ICU = intensive care unit; GCF = general care floor; RA = respiratory arrest.
