# Supplementary material for: Association of Opioids and Sedatives with Increased Risk of In-Hospital Cardiopulmonary Arrest from an Administrative Database
Source: PLoS One. 2016 Feb 25;11(2):e0150214. doi: 10.1371/journal.pone.0150214 (PMC4767404; doi:10.1371/journal.pone.0150214)
Supplement: S6 Table — (DOCX) [file pone.0150214.s006.docx]

**S6 Table. Opioid/*Sedative* Use, Location of Arrest, and Patient Acuity.**

|  | **Opioids +/- *Sedatives*** | | | | | | **Neither Opioids nor *Sedatives*** | | | | | |
| --- | --- | --- | --- | --- | --- | --- | --- | --- | --- | --- | --- | --- |
|  | **CPA/CPR** | | | **RA** | | | **CPA/CPR** | | | **RA** | | |
| **Characteristic** | **ICU**  (n=34,332) | **GCF**  (n=15,712) | **P**  **Value** | **ICU**  (n=1,598) | **GCF**  (n=1,272) | **P**  **Value** | **ICU**  (n=5,472) | **GCF**  (n=4,546) | **P**  **Value** | **ICU**  (n=191) | **GCF**  (n=219) | **P**  **Value** |
| Age <61 years | 10,899 (31.8) | 6,139 (39.1) | <0.0001 | 560 (35.0) | 448 (35.2) | 0.92 | 1,560 (28.5) | 1,037 (22.8) | <0.0001 | 60 (31.4) | 34 (15.5) | <0.0001 |
| CCI=0,1 | 8,773 (25.6) | 5,959 (37.9) | <0.0001 | 479 (30.0) | 460 (36.2) | 0.0004 | 1,845 (33.7) | 1,651 (36.3) | 0.007 | 66 (34.6) | 75 (34.3) | 0.95 |
| APR=1,2 | 1,807 (5.3) | 3,247 (20.7) | <0.0001 | 6 (0.4) | 25 (1.0) | <0.0001 | 539 (9.9) | 1,124 (24.7) | <0.0001 | 3 (1.6) | 10 (4.6) | 0.08 |
| Age <61 years *with* CCI=0,1  *and/or* APR=1,2 | 3,825 (11.1) | 3,683 (23.4) | <0.0001 | 227 (14.2) | 219 (17.2) | 0.03 | 798 (14.6) | 709 (15.6) | 0.16 | 30 (15.7) | 15 (6.9) | 0.004 |

Values presented as n (column %). APR = All Patient Refined Severity of Illness score (1 = Minor; 2 = Moderate); CCI = Charlson comorbidity index; CPA = Cardiopulmonary arrest; CPR = Cardiopulmonary resuscitation; GCF = General care floor; ICU = Intensive care unit; RA = Respiratory Arrest.
