# Supplementary material for: Association of Opioids and Sedatives with Increased Risk of In-Hospital Cardiopulmonary Arrest from an Administrative Database
Source: PLoS One. 2016 Feb 25;11(2):e0150214. doi: 10.1371/journal.pone.0150214 (PMC4767404; doi:10.1371/journal.pone.0150214)
Supplement: S7 Table — (DOCX) [file pone.0150214.s007.docx]

**S7 Table. Association between CPRA and Length of Hospital Stay and Cost of Care In Patients Receiving Opioids Projected Nationally in US.**

| **Variable** | **Projected National Number With CPRA (n=280,883)** | **Projected National Number Without CPRA (n=58,261,446)** | **Unadjusted Mean Difference** |
| --- | --- | --- | --- |
| **Hospital Stay** |  |  |  |
| Total LOS | 12.33 (17.9) | 4.90 (8.6) | 7.43 |
| ICU stay | 7.94 (10.7) | 3.79 (6.5) | 4.15 |
| **Hospital Cost** |  |  |  |
| Total hospital cost | $39,534 ($54,789) | $12,415 ($18,571) | $27,119 |
| Room and board cost | $16,236 ($26,074) | $4,596 ($8,046) | $11,639 |
| Surgery cost | $3,557 ($6,902) | $2,482 ($4,364) | $1,075 |
| Central supply cost | $4,618 ($9,439) | $2,544 ($8,353) | $2,074 |
| Anesthesia cost | $655 ($1,456) | $426 ($691) | $229 |
| Pharmacy cost | $5,026 ($11,541) | $1,141 ($6,807) | $3,885 |
| Other cost | $10,865 ($16,426) | $2,859 ($4,434) | $8,006 |
| **ICU cost** | $13,855 ($21,252) | $6,254 ($10,329) | $7,601 |

Values presented as mean (SD) days or $US. LOS = length of stay.
